# Results from a Pooled Analysis of Two European, Randomized, Placebo-Controlled, Phase 3 Studies of ATX-101 for the Pharmacologic Reduction of Excess Submental Fat

**DOI:** 10.1007/s00266-014-0364-9

**Published:** 2014-07-02

**Authors:** James McDiarmid, Jesus Benito Ruiz, Daniel Lee, Susanne Lippert, Claudia Hartisch, Blanka Havlickova

**Affiliations:** 1 McDiarmid-Hall Clinic, Derriford, Plymouth, UK; 2 Clínica Tres Torres, Barcelona, Spain; 3 KYTHERA Biopharmaceuticals, Inc., Calabasas, CA USA; 4 Global Clinical Development Dermatology, Bayer HealthCare, Sellerstrasse 31, 13353 Berlin, Germany

**Keywords:** Adipocytolysis, ATX-101, Deoxycholic acid, Injectable, Nonsurgical, Submental fat

## Abstract

**Background:**

The injectable adipocytolytic drug ATX-101 is the first nonsurgical treatment for the reduction of submental fat (SMF) to undergo comprehensive clinical evaluation. This study aimed to confirm the efficacy and safety of ATX-101 for SMF reduction through a post hoc pooled analysis of two large phase 3 studies.

**Methods:**

Patients with unwanted SMF were randomized to receive 1 or 2 mg/cm^2^ of ATX-101 or a placebo injected into their SMF during a maximum of four treatment sessions spaced approximately 28 days apart, with a 12-week follow-up period. The proportions of patients with reductions in SMF of one point or more on the Clinician-Reported SMF Rating Scale (CR-SMFRS) and the proportions of patients satisfied with the appearance of their face and chin [Subject Self-Rating Scale (SSRS) score ≥4] were reported overall and in subgroups. Other efficacy measures included improvements in the Patient-Reported SMF Rating Scale (PR-SMFRS), calliper measurements of SMF thickness, and assessment of skin laxity [Skin Laxity Rating Scale (SLRS)]. Adverse events and laboratory test results were recorded.

**Results:**

Significantly greater proportions of the patients had improvements in clinician-reported measures (≥1-point improvement in CR-SMFRS: 58.8 and 63.8 % of the patients who received ATX-101 1 and 2 mg/cm^2^, respectively, and 28.6 % of the placebo recipients;  *p* < 0.001 for both ATX-101 doses vs. placebo) and patient-reported measures (≥1-point improvement in PR-SMFRS: 60.0 and 63.1 % of the patients who received ATX-101 1 and 2 mg/cm^2^, respectively, vs. 34.3 % of the placebo recipients; *p* < 0.001 for both), analyzed alone or in combination, with ATX-101 versus placebo. These improvements correlated moderately with patient satisfaction regarding face and chin appearance (SSRS score ≥4: 60.8 and 65.4 % of the patients who received ATX-101 1 and 2 mg/cm^2^, respectively, vs. 29.0 % of the placebo recipients; *p* < 0.001 for both). In this study, ATX-101 was effective irrespective of gender, age, or body mass index. Reduction in SMF with ATX-101 was confirmed by calliper measurements (*p* < 0.001 for both doses vs. placebo) and generally did not lead to worsening of skin laxity (SLRS improved or was unchanged: 91.3 and 90.5 % of the patients who received ATX-101 1 and 2 mg/cm^2^, respectively, and 91.6 % of the placebo recipients). Adverse events were mostly transient, mild to moderate in intensity, and localized to the treatment area.

**Conclusion:**

The findings show ATX-101 to be an effective and well-tolerated pharmacologic treatment for SMF reduction.

**Level of Evidence I:**

This journal requires that authors assign a level of evidence to each article. For a full description of these Evidence-Based Medicine ratings, please refer to the Table of Contents or the online Instructions to Authors www.springer.com/00266.

## Introduction

Patients with unwanted submental fat (SMF) are frequently dissatisfied with the appearance of their face and chin [1]. This preplatysmal subcutaneous fat that accumulates in the submental compartment leads to loss of mandibular line definition and a perception of an aging or overweight appearance [2, 3].

Unwanted SMF can be effectively addressed by liposuction or as part of a surgical procedure such as a face-lift [2, 4]. However, not all patients are suitable for surgery, and others may be concerned about undergoing an invasive procedure. For these patients, a nonsurgical alternative is warranted.

Nonsurgical techniques for localized fat reduction comprise nonsurgical energy devices such as external laser, radiofrequency, cryolipolysis, and ultrasound [5–7], and injectable fat-reducing formulations [8]. The latter have been investigated for the reduction of subcutaneous fat in small studies [9–16], but no injectable pharmacologic treatment is currently licensed for the reduction of SMF. Overall, robust clinical evidence regarding the efficacy and safety of nonsurgical methods for the reduction of SMF is lacking.

Studies have shown the endogenous molecule deoxycholic acid to be the active lytic agent in previous injectable formulations [17–19]. As a proprietary, synthetically derived, purified formulation of deoxycholic acid, ATX-101 causes localized adipocytolysis when injected into subcutaneous fat. Histologic evidence indicates that disruption of adipocyte membranes by deoxycholate/deoxycholic acid prompts a mild inflammatory tissue response, which is responsible for clearing the cellular debris through macrophage recruitment and subsequent phagocytosis [20–23].

In healthy subjects, ATX-101-induced adipocytolysis was not associated with a significant increase in plasma lipid levels over time [23]. In addition, ATX-101 showed no accumulation at the injection site because of rapid clearance into the enterohepatic circulation. Nonspecific protein binding of deoxycholic acid was found to limit cellular membrane breakdown in protein-rich tissues such as muscle and skin, thus favoring lysis of fat cells, which are protein poor [22].

Currently, ATX-101 is under investigation as a novel nonsurgical intervention for the reduction of unwanted SMF through a rigorously evaluated and comprehensive clinical trial program. In two pivotal European randomized phase 3 clinical studies, ATX-101 was effective and well tolerated for the reduction of unwanted SMF [24, 25]. A post hoc analysis of the pooled data from these studies was conducted to confirm the efficacy and safety outcomes in the large combined patient population. The large number of patients also allowed for exploratory analysis of outcomes in relevant subgroups. The results are presented in this report.

## Methods

### Study Design

Two phase 3, randomized, double-blind, placebo-controlled clinical studies (NCT01305577 and NCT01294644) designed to test the superiority of ATX-101 over a placebo were conducted in 57 centers in Germany, France, Spain, Italy, Belgium, and the UK. The studies were conducted with the overview and approval of ethics boards and under the terms of the Declaration of Helsinki and the International Conference on Harmonisation Guideline E6: Good Clinical Practice. All the patients provided written, informed consent to participate in the study, and all local legal and regulatory stipulations were followed.

### Patients and Enrollment Criteria

**Fig. 1:**
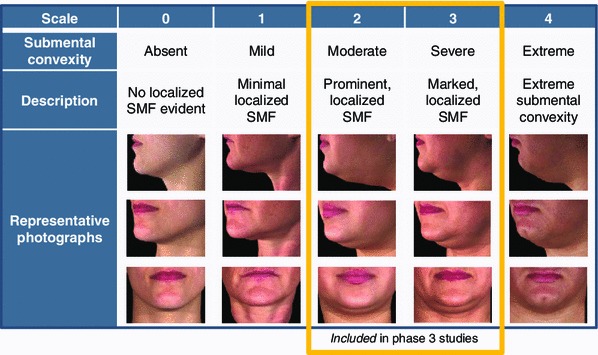
Clinician-Reported Submental Fat Rating Scale (CR-SMFRS)

The patients were screened at the first study visit and considered eligible to participate in the studies if they had moderate or severe submental convexity and prominent to marked localized SMF, as judged by the investigator. This corresponded to Clinician-Reported SMF Rating Scale (CR-SMFRS) grade 2 or 3 on a scale of 0 (no submental convexity or evidence of localized SMF) to 4 (extreme submental convexity; Fig. 1).

In addition, the patients were included if their degree of satisfaction with the appearance of their face and chin corresponded to a score of 0 (extremely dissatisfied) to 3 (neither satisfied nor dissatisfied) on a scale of 0 to 6 (extremely satisfied) on the Subject Self-Rating Scale (SSRS). The patients also were required to be 18–65 years of age inclusive, to have a body mass index (BMI) of 30 kg/m^2^ or less, and to have demonstrated stable body weight for at least the previous 6 months. The principal exclusion criteria were a history of SMF treatment or other recent aesthetic treatment of the chin or neck, the presence of loose skin or previous trauma in the neck or chin area, or prominent platysmal bands. In addition, the patients were required not to change dietary or exercise practices or start a weight-reduction regimen during the course of the study. All patients with concomitant disease that could potentially interfere with the study treatment or its outcomes were excluded from the study. Women of reproductive potential were required to practice birth control during the study and were excluded if pregnant or lactating.

### Randomization and Treatment

After screening at study visit 1, baseline evaluations and randomization were performed in a 1:1:1 ratio at visit 2. Patients were allocated a unique randomization number via a computerized Web/voice-response system that corresponded to an ATX-101 (1 or 2 mg/cm^2^) or placebo treatment kit, each of which had an identical appearance and a blinded label. The computerized system was provided by a third party, and both the patients and the investigators were unaware of the random allocation sequence, which was stratified by center.

The patients received one of two ATX-101 dosing regimens (1 or 2 mg/cm^2^) or a placebo (sodium phosphate and sodium chloride in water for injection) administered in an identical fashion in up to four treatment sessions separated by approximately 28 days (visits 2–5). At each treatment session, the patients received not more than 50 injections of ATX-101 or placebo into the preplatysmal SMF. The injections were spaced evenly at 1-cm intervals by use of a grid, and a maximum of 0.2 ml per injection was administered up to a maximum of 10 ml per treatment session. Topical anesthesia could be given if judged necessary. The patients could receive fewer than the maximum of four treatment sessions for safety reasons, at the patient’s request, or because the clinician or patient judged that therapeutic success had been achieved. The patients returned for two follow-up visits 4 weeks (visit 6) and 12 weeks (visit 7) after the final treatment session.

### Outcome Measures

Efficacy outcomes were evaluated in the intention-to-treat population, which comprised all randomized patients who had at least one efficacy assessment (CR-SMFRS or SSRS) at baseline (Table 1). The co-primary efficacy end points were an improvement of one point or more from baseline rated by the clinician using the CR-SMFRS and a final score of 4 (slightly satisfied) to 6 (extremely satisfied) rated by the patient using the SSRS, indicating satisfaction with the appearance of the face and chin. In the pooled analysis, the primary efficacy outcomes were investigated in prespecified subgroups of gender, age, and BMI category.

**Table 1:** Outcomes measures used in the study for which data are presented

Efficacy outcomes	Evaluator	Outcome measures	Method of evaluation	Rating scale (range)
Primary	Physician	SMF severity (submental convexity and amount of SMF)	CR-SMFRS	0 (No submental convexity–no localized fat) 1 (Mild submental convexity, minimal localized SMF) 2 (Moderate submental convexity, prominent localized SMF) 3 (Severe submental convexity, marked localized SMF) 4 (Extreme submental convexity)
Primary	Patient	Satisfaction with appearance in association with the face and chin	SSRS	0 (Extremely dissatisfied) 1 (Dissatisfied) 2 (Slightly dissatisfied) 3 (Neither satisfied nor dissatisfied) 4 (Slightly satisfied) 5 (Satisfied) 6 (Extremely satisfied)
Secondary	Physician	SMF thickness	Calliper measurement	SMF thickness measured in millimeters
Secondary	Patient	SMF severity (amount of SMF)	PR-SMFRS	No chin fat at all A slight amount of chin fat A moderate amount of chin fat A large amount of chin fat A very large amount of chin fat
Other	Physician	Skin laxity	SLRS	1 (No laxity) 2 (Minimal laxity) 3 (Moderate laxity) 4 (Very lax)

*SMF* submental fat, *CR-SMFRS* Clinician-Reported Submental Fat Rating Scale, *SSRS* Subject Self-Rating Scale, *PR-SMFRS* Patient-Reported Submental Fat Rating Scale, *SLRS* Skin Laxity Rating Scale

Secondary and other efficacy outcomes (Table 1) included changes in skin laxity from baseline, assessed by clinicians using the Skin Laxity Rating Scale (SLRS), as well as changes in CR-SMFRS scores and calliper measurements of SMF thickness over the course of treatment. Patient-reported secondary and other outcomes included an improvement of one point or more from baseline using the Patient-Reported SMF Rating Scale (PR-SMFRS), which assessed SMF severity from a patient perspective. These scales were used in phase 2 and 3 studies of ATX-101 treatment in the USA [26–29]. In these and other observational studies (data on file), psychometric properties were found to be appropriate for assessment of the outcomes. Additional patient-reported measures of outcomes were assessed but are not discussed in this report.

As post hoc analyses, the proportion of responders to treatment (≥1-point reduction on the CR-SMFRS) and the proportion of patients satisfied with their appearance in association with their face and chin (SSRS score ≥4) were documented for both the overall pooled population and the subgroup populations according to gender, age, and BMI category. The proportions of patients with an improvement of two points or more from baseline on the CR-SMFRS and improvements of at least one point and of at least two points from baseline on the PR-SMFRS also were recorded.

In the pooled analysis, the proportions of patients for whom a clinician-rated improvement of at least one point or at least two points (CR-SMFRS) was simultaneously accompanied by an equivalent improvement in patient rating (PR-SMFRS) also were analyzed. In addition, the correlations between the clinician- and patient-reported ratings of SMF severity (CR-SMFRS and PR-SMFRS) and the patient-reported ratings of satisfaction with the appearance of their face and chin after treatment (SSRS) were investigated.

The safety population included all the patients who received at least one treatment. Adverse events, recorded at each visit and approximately 7 days after each treatment session, were characterized descriptively in terms of association with treatment, by the day of onset and cessation, and by severity and intensity. Changes in clinical laboratory parameters and other tests as well as variations in vital signs, body temperature, and body weight, were evaluated throughout the study.

### Statistical Analysis

The two phase 3 studies had an identical design and were conducted in parallel at different centers, which allowed for post hoc pooling of the data. This was done by adding the data sets without further modification. Each individual study was designed with a 90 % power to detect differences between the ATX-101 treatment groups and the placebo group. Due to a greater number of patients, the pooled analysis had an increased statistical power compared with each study alone. The results of these post hoc analyses are for exploratory purposes only.

Full statistical methods have been reported previously [24, 25]. Statistical comparisons of efficacy between ATX-101 and placebo were made using a two-sided test with a type 1 error rate of 0.05, and missing values were imputed by carrying the last observation forward. The proportions of responders, as assessed by CR-SMFRS, PR-SMFRS, and SSRS, were analyzed by binary logistic regression, with posttreatment and baseline values as cofactors.

Conclusions for the percentage of responders in the subgroups (gender, age, BMI category) were drawn using odds ratios (ORs) derived directly from the response rates, with 95 % confidence intervals (CIs). Changes in calliper measurements from baseline were analyzed at each post-baseline visit as the dependent variable by a repeated measurement analysis of covariance. Demographic and clinical parameters were analyzed descriptively. Adverse events (number of patients and number of events) were categorized by association with treatment, study withdrawal, death, severity, intensity, system organ class, and preferred term. Statistical summaries were based on adverse events emerging after the start of treatment.

## Results

### Patient Demographics

Of the 723 patients randomized across the two studies, 716 were treated with either ATX-101 or placebo and formed the safety population for the pooled analysis. Two patients who were randomized had no baseline efficacy assessment. Therefore, the pooled intention-to-treat population comprised 721 patients.

The demographic characteristics of the safety population are provided in Table 2. The majority of the patients were female (74 %), older than 30 years (92 %), and Caucasian (94 %), with a mean BMI of 26 kg/m^2^. The characteristics were well balanced between the treatment groups.

**Table 2:** Patient baseline demographics (intention-to-treat population)

Variables	Placebo (*n* = 238)	ATX-101 1 mg/cm^2^ (*n* = 240)	ATX-101 2 mg/cm^2^ (*n* = 243)	Overall (*n* = 721)	*p* Value^a^
Female: *n* (%)	167 (70.2)	186 (77.5)	183 (75.3)	536 (74.3)	0.170
Mean age ± SD (years)	46.3 ± 9.91	45.9 ± 10.53	46.3 ± 9.85	46.2 ± 10.09	0.873
Race: *n* (%)					
Caucasian	223 (93.7)	224 (93.3)	230 (94.7)	677 (93.9)	0.741
Other	15 (6.3)	16 (6.7)	13 (5.3)	44 (6.1)	
BMI (kg/m^2^), mean ± SD	25.8 ± 2.67	26.1 ± 2.73	26.1 ± 2.90	26.0 ± 2.77	0.445

*BMI* body mass index

^a^ *p* Values refer to differences between categories from a Pearson’s *χ* ^2^ test

**Fig. 2:**
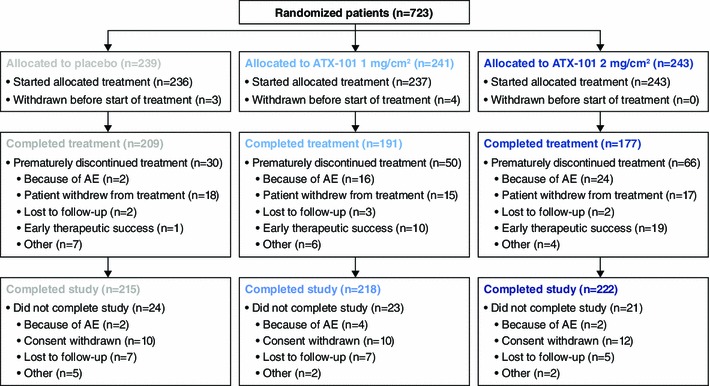
Disposition of all the randomized patients. The patients classified as “prematurely discontinued treatment” still completed the study by completing the follow-up visits. The patients classified as “did not complete study” did not complete the follow-up visits. *AE* adverse event

A study flow diagram is shown in Fig. 2. Approximately 80 % of the patients completed the four planned treatment sessions (87 and 73 % for ATX-101 1 and 2 mg/cm^2^, respectively, and 87 % for the placebo). In the ATX-101 groups, premature treatment discontinuation was attributable to early therapeutic success (≥1-point improvement in CR-SMFRS) in 4 and 8 % of the patients treated with ATX-101 1 and 2 mg/cm^2^, respectively, versus less than 1 % of those who received the placebo. This was reflected in the total volume of injections received (16.0 ml for ATX-101 1 mg/cm^2^, 13.6 ml for ATX-101 2 mg/cm^2^, and 18.0 ml for placebo). Adverse events led to treatment discontinuation in 7 and 10 % of the patients in the ATX-101 1 and 2 mg/cm^2^ groups, respectively, compared with 1 % of the placebo recipients. More than 90 % of the patients completed the study in which they were enrolled.

### Primary Efficacy Outcomes

**Fig. 3:**
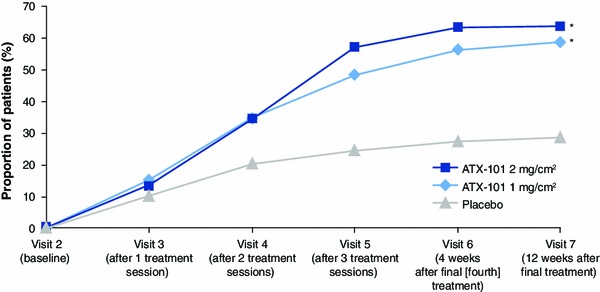
Proportion of treatment responders (≥1-point reduction on the CR-SMFRS) from visit 2 (baseline) to the final follow-up visit (12 weeks after the final treatment). Intention-to-treat population at study visit 7, with the last observation carried forward for patients with missing data. *p < 0.001 versus placebo (Bonferroni–Holm testing procedure). *CR-SMFRS* Clinician-Reported Submental Fat Rating Scale

The co-primary efficacy end points were achieved for both ATX-101 doses. The primary clinician-rated efficacy outcome (≥1-point improvement in CR-SMFRS from baseline 12 weeks after the final treatment) occurred for significantly more patients who received ATX-101 than for those who received the placebo (58.8 % for ATX-101 1 mg/cm^2^, 63.8 % for ATX-101 2 mg/cm^2^, and 28.6 % for the placebo; Fig. 3). This corresponded to ORs of treatment response with ATX-101 of 3.5 (95 % CI 2.4–5.1) and 4.4 (95 % CI 3.0–6.4) for each ATX-101 dose, respectively, compared with the placebo (*p* < 0.001 for both comparisons).

**Fig. 4:**
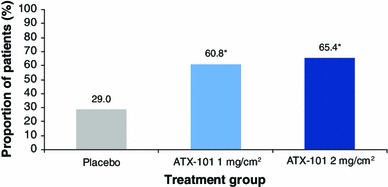
Proportion of patients satisfied with their appearance in association with their face and chin (SSRS score ≥4) at the final follow-up visit (12 weeks after the final treatment). Intention-to-treat population at study visit 7, with the last observation carried forward for patients with missing data. *p < 0.001 versus placebo (Bonferroni–Holm testing procedure). *SSRS* Subject Self-Rating Scale

**Fig. 5:**
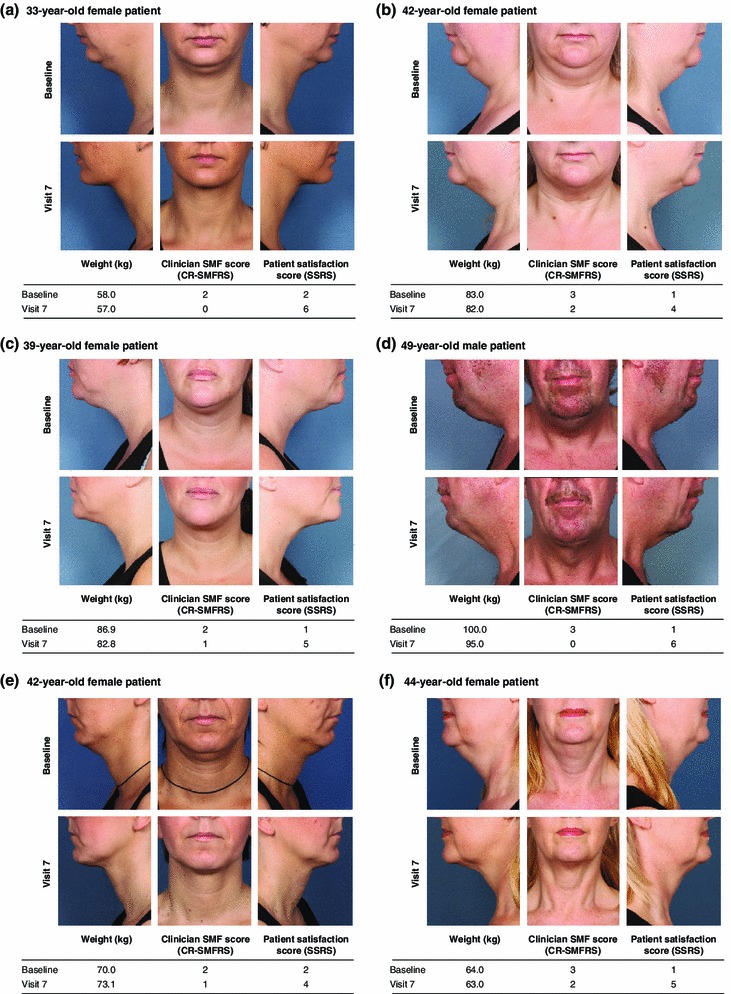
Selected patient images before and after treatment with ATX-101 2 mg/cm^2^ (**a**–**d**) and ATX-101 1 mg/cm^2^ (**e**, **f**). **a**, **e** Reproduced from [25]. **b**, **c** Reproduced from [24]. Patient photographs published with permission. *CR-SMFRS* Clinician-Reported Submental Fat Rating Scale, *SSRS* Subject Self-Rating Scale

The primary patient-reported outcome (SSRS score ≥4, indicating satisfaction with the appearance of the face and chin) also occurred for significantly more patients who received ATX-101 than for those who received the placebo (60.8 and 65.4 % for ATX-101 1 and 2 mg/cm^2^, respectively, vs. 29 % for the placebo; Fig. 4). The ORs of treatment response based on the SSRS score for ATX-101 versus placebo were 4.0 (95 % CI 2.7–5.9) and 4.8 (95 % CI 3.3–7.1), respectively (*p* < 0.001 for both comparisons). Example patient photographs are provided in Fig. 5.

In this pooled analysis, the influence of gender, age, and BMI category on treatment efficacy (≥1-point reduction in CR-SMFRS) and satisfaction of the patients with their face and chin appearance (SSRS score ≥4) was evaluated in an attempt to identify a particular profile of patients more likely to benefit from treatment with ATX-101. The outcomes for the primary efficacy end points in these subpopulations of interest were broadly consistent with those in the overall pooled population and consistently favored ATX-101 over placebo, generally independent of gender, increasing age, or BMI score (data not shown). The responses of female patients in terms of CR-SMFRS (≥1-point improvement) and SSRS (score ≥4) were superior to those in the placebo group for both ATX-101 doses, whereas among male patients, the 2 mg/cm^2^ dose was superior to the placebo, but not the 1 mg/cm^2^ dose. The responses were consistently superior with ATX-101 compared with the placebo for the different age groups, except for the patients 18–30 years of age receiving ATX-101 1 mg/cm^2^, in which the difference did not reach statistical significance. Both ATX-101 doses were superior to the placebo in all BMI categories.

### Secondary and Other Efficacy Outcomes

In the pooled population, skin laxity (assessed using the SLRS) was improved or unchanged in 91.3 % of the patients who received ATX-101 1 mg/cm^2^, 90.5 % of those who received ATX-101 2 mg/cm^2^, and 91.6 % of the placebo recipients. Although a low proportion of the patients in all the groups experienced a worsening of skin laxity after treatment (8.8 and 9.5 % for ATX-101 1 and 2 mg/cm^2^, respectively, vs. 8.4 % for the placebo), the patients receiving ATX-101 1 mg/cm^2^ (30.0 %) and 2 mg/cm^2^ (21.6 %) showed a greater tendency for skin laxity improvement than the placebo group (13.6 %).

The proportion of patients achieving a CR-SMFRS response increased over the course of treatment with ATX-101 and was noticeably better than with the placebo by the third treatment session (Fig. 3). Calliper measurements, used as an objective tool to measure reductions in SMF thickness, reflected this same trend, and 12 weeks after the final treatment, statistically significant reductions occurred with each ATX-101 dose compared with the placebo (−1.29 mm [95 % CI −1.90 to −0.68] and −1.52 mm [95 % CI −2.13 to −0.91] for ATX-101 1 and 2 mg/cm^2^, respectively; *p* < 0.001 for both doses vs. placebo).

**Fig. 6:**
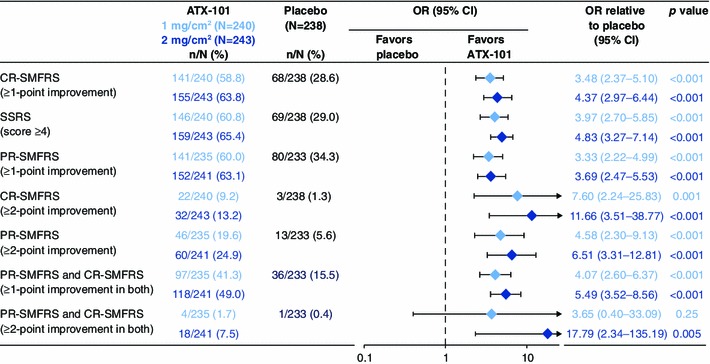
Outcomes and odds ratios (ORs) for ATX-101 1 and 2 mg/cm^2^, and placebo for primary and secondary clinician- and patient-reported efficacy end points. The* graphic* representation of ORs shows superiority of ATX-101 over placebo when the 95 % confidence interval (CI) lies completely on the *right-hand side* of the *dotted vertical line*. The ORs and *p* values were determined by binary logistic regression. The intention-to-treat population at study visit 7, with last observation carried forward for patients with missing data, is shown. *CI* confidence interval, *CR-SMFRS* Clinician-Reported Submental Fat Rating Scale, *PR-SMFRS* Patient-Reported Submental Fat Rating Scale, *SSRS* Subject Self-Rating Scale

Consistent with these measures, significantly more patients reported an improvement of one point or more in their SMF (PR-SMFRS) with ATX-101 (60.0 and 63.1 % with ATX-101 1 and 2 mg/cm^2^, respectively) than with the placebo (34.3 %; *p* < 0.001 for both ATX-101 doses; Fig. 6). Significantly more patients who received ATX-101 1 mg/cm^2^ (41.3 %) and 2 mg/cm^2^ (49.0 %) than placebo recipients (15.5 %) had both a clinician- and patient-evaluated improvement of one point or more on the CR-SMFRS and PR-SMFRS (*p* < 0.001 for both ATX-101 doses; Fig. 6).

An improvement of two points or more in the CR-SMFRS score was recorded for significantly more patients in the ATX-101 groups than in the placebo group 12 weeks after the final treatment (9.2 and 13.2 % for ATX-101 1 and 2 mg/cm^2^, respectively, vs. 1.3 % for placebo; *p* ≤ 0.001). This was also the case for improvements of two points or more in the PR-SMFRS score (19.6 % with ATX-101 1 mg/cm^2^, 24.9 % with ATX-101 2 mg/cm^2^, and 5.6 % with the placebo; *p* < 0.001 for both ATX-101 doses). A significantly greater proportion of patients who received the ATX-101 2 mg/cm^2^ dose had improvements of two points or more simultaneously in both the CR-SMFRS and PR-SMFRS scores than the patients who received the placebo (7.5 vs. 0.4 %; *p* = 0.005), but the difference was not significant for the lower ATX-101 dose (Fig. 6).

Overall, there was a moderate, positive correlation in terms of treatment response between the patient-reported SSRS score (satisfaction with the appearance of the face and chin) and the amount of SMF (PR-SMFRS; correlation coefficient 0.36, in which a value between 0.30 and 0.49 is commonly considered to represent a moderate correlation), and between the SSRS score and the clinician evaluation of SMF size and convexity (CR-SMFRS; correlation coefficient 0.37).

The correlation between the patient rating of SMF severity (PR-SMFRS) and the CR-SMFRS was slightly lower than for the aforementioned comparisons (correlation coefficient 0.28). For up to 70 % of the patients, there was a concordance between the clinician and patient assessments of response or nonresponse to treatment based on CR-SMFRS, PR-SMFRS, and SSRS scores.

### Safety Outcomes

Treatment-emergent adverse events, for which investigators made a blinded attribution of relationship to the study treatment, occurred in 95.6 % of the ATX-101 recipients and 55.5 % of the placebo recipients. All the treatment-related adverse events were associated with the treatment area, and those that occurred most frequently in a comparison of the combined incidences for both ATX-101 doses with the placebo were pain (84.6 vs. 27.5 %), swelling including edema (60.6 vs. 26.3 %), bruising including bleeding (56.0 vs. 45.3 %), numbness (49.0 vs. 2.1 %), erythema (40.2 vs. 22.5 %), and induration including fibrosis (20.0 vs. 1.7 %; Table 3). Most of these events were mild or moderate in intensity.

**Table 3:** Treatment-emergent adverse events at the injection site judged to be related to treatment

Adverse event by injection-site category	Incidence *n* (%)			
	Placebo (*n* = 236)	ATX-101 1 mg/cm^2^ (*n* = 237)	ATX-101 2 mg/cm^2^ (*n* = 243)	ATX-101 total^a^ (*n* = 480)
Pain including burning	65 (27.5)	199 (84.0)	207 (85.2)	406 (84.6)
Swelling including edema	62 (26.3)	144 (60.8)	147 (60.5)	291 (60.6)
Bruising including bleeding	107 (45.3)	137 (57.8)	132 (53.4)	269 (56.0)
Numbness	5 (2.1)	109 (46.0)	126 (51.9)	235 (49.0)
Erythema	53 (22.5)	96 (40.5)	97 (40.0)	193 (40.2)
Induration including fibrosis	4 (1.7)	40 (16.9)	56 (23.0)	96 (20.0)

Incidence ≥10 % for either ATX-101 or placebo; safety population

^a^Incidences for ATX-101 1 and 2 mg cm^2^ combined

Except for pain, the injection-site adverse events in the aforementioned categories were reported to have severe intensity by less than 10 % of the patients receiving ATX-101. Approximately 60 % of the ATX-101 recipients reported moderate or severe injection-site pain, but this adverse event had a median duration of only 1 day (mean ± standard deviation [SD], 6.3 ± 14.02 days for ATX-101 1 mg/cm^2^ and 6.5 ± 15.44 days for ATX-101 2 mg/cm^2^).

**Fig. 7:**
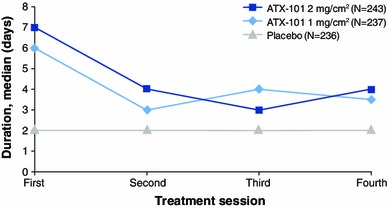
Median duration of adverse events occurring in the treatment area by treatment visit (safety population). See the main text for the corresponding mean values

Most of the injection-site adverse events resolved in the interval between treatment sessions (28 days). The duration of the injection-site adverse events was longer after the first treatment session (median 6–7 days, mean ± SD, 15.2 ± 25.80 days for ATX-101 1 mg/cm^2^ and 19.3 ± 31.89 days for ATX-101 2 mg/cm^2^) than after the subsequent sessions (median 3–4 days, mean ~8–13 days, Fig. 7). In total, 5.5 % of the patients in the placebo group, 8.0 % of those in the ATX-101 1 mg/cm^2^ group, and 9.5 % of those in the ATX-101 2 mg/cm^2^ group had an event recorded as unresolved at the end of the study follow-up period. All these events were subsequently reported to have resolved.

Five cases of injection-site nerve injury occurred (2.1 %), all with the higher ATX-101 dose. One of these cases was considered to be a treatment-related serious adverse event (temporary asymmetric smile possibly associated with injury to the branch of the marginal mandibular nerve on the right side of the face). No hospitalization or further treatment was required as a result of this event, which subsequently resolved without sequelae. The nonserious nerve injury events all resolved within the study period. No relevant changes in clinical laboratory tests or vital signs were observed during the study, and no deaths occurred.

## Discussion

In this pooled analysis, treatment with ATX-101 was associated with up to 4.4-fold greater likelihood (based on ORs) of a reduction in clinician-rated SMF severity (CR-SMFRS) and was up to 4.8-fold more likely to result in satisfaction of the patients with the appearance of their face and chin after treatment (SSRS) compared with the placebo. As in each individual phase 3 study and consistent with efficacy and tolerability observations in previous phase 1 and 2 studies [23, 26–32], the outcomes for the primary efficacy end points with both ATX-101 doses tested were clinically and statistically superior to the placebo outcomes. The response to treatment with the ATX-101 2 mg/cm^2^ dose overall was empirically better, and a greater proportion of the patients who received this higher dose required fewer than the maximum of four treatment sessions to achieve an improvement of one point or more in CR-SMFRS score compared with those who received the lower dose (ATX-101 1 mg/cm^2^). Efficacy was broadly consistent across the subgroups of gender, age and BMI category studied, suggesting that ATX-101 may be suitable for a broad population of patients.

Although a trend toward a treatment effect was observed, the statistical significance with ATX-101 1 mg/cm^2^ compared with the placebo was not achieved for male patients or patients 18–30 years of age. This could simply be the result of the relatively low patient numbers in these subgroups, could reflect the presence of other confounding factors, or could suggest a need for more intensified treatment of these patients. In terms of the primary end points in both of these subgroups, ATX-101 2 mg/cm^2^ was superior to the placebo.

The results of the secondary efficacy outcomes were consistent with those from the individual phase 3 studies [24, 25]. The pooled analysis allowed for comparison between ATX-101 and the placebo for other end points, such as an improvement of two points or more in the clinician and patient rating scales of SMF severity (CR-SMFRS and PR-SMFRS). The ATX-101 treatment at both 1 and 2 mg/cm^2^ was associated with a significantly greater proportion of patients who achieved this level of response on either scale.

When the CR-SMFRS and PR-SMFRS scales were considered in tandem, the findings showed that both ATX-101 doses were associated with a significantly greater proportion of improvements of one point or more in both scales compared with the placebo. This also was the case for improvements of two points or more with the ATX-101 2 mg/cm^2^ dose. Clinician-reported improvements in SMF convexity and severity (CR-SMFRS) had moderate positive correlations with patient evaluations of SMF severity (PR-SMFRS) and satisfaction with the appearance of the face and chin after treatment (SSRS), indicating that the patient-reported outcome measures provided an important and valid means of assessing ATX-101 treatment efficacy and comprehensively complemented the clinician-reported outcome measures.

The ATX-101 treatment was well tolerated and mainly associated with transient reactions in the treatment area of the type that might be expected with an injectable adipocytolytic therapy and the subsequent tissue response. The most common reactions (pain, swelling, bruising, numbness, erythema, and induration) generally resolved in the 28-day interval between treatment sessions. For ATX-101, the median duration of adverse events was shorter for the second, third, and fourth treatment sessions than for the first session. This may reflect increased patient familiarity with the injections as well as a decrease in the amount of cell lysis and tissue response with later treatments because of diminished SMF.

Most of the adverse events with ATX-101 were mild or moderate in intensity. Injection-site pain was more commonly rated as moderate or severe but resolved within a median time of 1 day. Of the few nerve injury adverse events reported, only one was considered a serious adverse event related to treatment, and all such events resolved without sequelae. These events were possibly the result of injections administered too deeply into the platysma muscle or too close to the marginal mandibular nerve, emphasizing the importance of proper injection location and technique and of good knowledge concerning the relevant anatomy and anatomic landmarks.

From the patient’s perspective, the overall recovery period associated with ATX-101 injections appears to be acceptable compared with currently available interventions for SMF reduction and may be similar to that for localized liposuction. The normal recovery period for the latter is 3–4 weeks [33]. However, for some liposuction procedures in exceptional circumstances, full recovery can take up to 1 year [34].

Currently, liposuction, with or without the use of an accompanying energy device, is the most commonly used intervention for SMF reduction [5, 35]. Liposuction is suitable for patients who require localized fat removal. Those who desire more comprehensive facial remodeling are likely to be candidates for face-lifts or other more specialized surgical techniques. However, although liposuction is considerably less invasive than surgery, some patients may still be disinclined to undergo this procedure. For others, the surgeon may be concerned about the potential for unfavorable aesthetic results, such as postoperative anterior platysmal banding [4, 36]. For such patients, whose treatment goal is to improve the submental profile without further facial remodelling, ATX-101 may provide a minimally invasive option that offers a clinically meaningful improvement in chin profile and is well tolerated.

This pooled analysis was associated with some limitations. In a previous phase 2 study of ATX-101, magnetic resonance imaging (MRI) was used for objective assessment of reductions in SMF and demonstrated a significant treatment effect compared with placebo [37]. However, it was not considered practical to use MRI in these large, clinic-based phase 3 trials. Therefore, calliper measurements were used as an objective tool to measure reductions in SMF thickness. A further limitation was the post hoc nature of this analysis, although this must be considered in the light of the prospective and robust nature of the two individual studies, their positive results, and the increased statistical power of the pooled data set.

The injectable adipocytolytic drug ATX-101 is the first nonsurgical treatment for SMF reduction to undergo comprehensive clinical evaluation in a large sample of patients. The results of two phase 3 studies, as confirmed by this pooled analysis, demonstrated that ATX-101 was effective based on clinician, patient, and objective measures and was well tolerated. For patients for whom a minimally invasive procedure is appropriate, ATX-101 may provide a relatively simple, injectable approach to the reduction of unwanted SMF.
